# Exploration of Pharmacological Mechanisms of Dapagliflozin against Type 2 Diabetes Mellitus through PI3K-Akt Signaling Pathway based on Network Pharmacology Analysis and Deep Learning Technology

**DOI:** 10.2174/0115734099274407231207070451

**Published:** 2024-01-09

**Authors:** Jie Wu, Yufan Chen, Shuai Shi, Junru Liu, Fen Zhang, Xingxing Li, Xizhi Liu, Guoliang Hu, Yang Dong

**Affiliations:** 1 Department of Cardiology, Jinhua People's Hospital, Jinhua, Zhejiang, China;; 2 Department of Blood Donation Service, Central Blood Station of Jinhua, Jinhua, Zhejiang, China;; 3 Department of IVF, Jinhua People's Hospital, Jinhua, Zhejiang, China;; 4 Department of Endocrinology, Jinhua People's Hospital, Jinhua, Zhejiang, China;; 5 Department of Ultrasound in Medicine, Jinhua People's Hospital, Jinhua, Zhejiang, China

**Keywords:** Type 2 diabetes mellitus, dapagliflozin, network pharmacology, deep learning, PI3K-Akt signaling pathway, pharmacological mechanism

## Abstract

**Background:**

Dapagliflozin is commonly used to treat type 2 diabetes mellitus (T2DM). However, research into the specific anti-T2DM mechanisms of dapagliflozin remains scarce.

**Objective:**

This study aimed to explore the underlying mechanisms of dapagliflozin against T2DM.

**Methods:**

Dapagliflozin-associated targets were acquired from CTD, SwissTargetPrediction, and SuperPred. T2DM-associated targets were obtained from GeneCards and DigSee. VennDiagram was used to obtain the overlapping targets of dapagliflozin and T2DM. GO and KEGG analyses were performed using clusterProfiler. A PPI network was built by STRING database and Cytoscape, and the top 30 targets were screened using the degree, maximal clique centrality (MCC), and edge percolated component (EPC) algorithms of CytoHubba. The top 30 targets screened by the three algorithms were intersected with the core pathway-related targets to obtain the key targets. DeepPurpose was used to evaluate the binding affinity of dapagliflozin with the key targets.

**Results:**

In total, 155 overlapping targets of dapagliflozin and T2DM were obtained. GO and KEGG analyses revealed that the targets were primarily enriched in response to peptide, membrane microdomain, protein serine/threonine/tyrosine kinase activity, PI3K-Akt signaling pathway, MAPK signaling pathway, and AGE-RAGE signaling pathway in diabetic complications. AKT1, PIK3CA, NOS3, EGFR, MAPK1, MAPK3, HSP90AA1, MTOR, RELA, NFKB1, IKBKB, ITGB1, and TP53 were the key targets, mainly related to oxidative stress, endothelial function, and autophagy. Through the DeepPurpose algorithm, AKT1, HSP90AA1, RELA, ITGB1, and TP53 were identified as the top 5 anti-targets of dapagliflozin.

**Conclusion:**

Dapagliflozin might treat T2DM mainly by targeting AKT1, HSP90AA1, RELA, ITGB1, and TP53 through PI3K-Akt signaling.

## 1 INTRODUCTION

Diabetes mellitus (DM) is a prevalent endocrine disorder identified by unusually high blood sugar levels, forecasted to afflict 693 million adults globally by 2045 [1]. Furthermore, DM is believed to be responsible for 11.3% of global deaths [2]. Consequently, the escalating prevalence and mortality rate have significantly burdened the healthcare infrastructure [3]. More than 90% of all patients with DM reportedly suffer from type 2 diabetes mellitus (T2DM) [4]. T2DM is a metabolic condition characterizedcharacterized by elevated blood sugar and insulin resistance [5]. T2DM can lead to various complications, such as retinopathy, neuropathy, nephropathy, and cardiovascular disease [6]. There are multiple hypoglycemic drugs available for T2DM treatment, such as sulfonylureas, meglitinides, biguanides, and glucagon-like peptide-1 receptor agonists [7]. Commonly, metformin is employed as the first-line medication for T2DM [8]. However, strategies to control hyperglycemia by elevating glucose uptake (insulin, thiazolidinediones, and biguanides) or augmenting the production of the net insulin (sulfonylureas) have shown limited benefits [9].

Sodium-glucose cotransporter-2 (SGLT2) inhibitors represent a relatively novel category of antihyperglycaemic agents (AHAs) utilized for managing T2DM by impeding glucose reuptake in the renal proximal tubules [10, 11]. Different from other hypoglycemic drugs, SGLT2 inhibitors eliminate glucose from the body, consequently diminishing systemic and cellular glucose toxicity [11]. Besides glucose-reducing capacity, SGLT2 inhibitors also contribute to weight loss, particularly in the abdominal region, blood pressure reduction, as well as enhancement of lipid profile and serum uric acid levels [12]. Dapagliflozin, a reversible and highly selective SGLT2 inhibitor, was the first selective SGLT2 inhibitor to be approved for T2DM treatment in 2012 [9, 13]. Some large-scale clinical studies have revealed the beneficial effects of dapagliflozin on the amelioration of cardiovascular outcomes in patients with T2DM [14-16]. However, the specific molecular mechanisms of dapagliflozin against T2DM are still poorly understood.

Over the recent years, network pharmacology has emerged as a novel field drawing on the principles of systems biology and multi-target pharmacology. This discipline efficiently conducts predictive analyses of drug action mechanisms, identifies novel drug targets, and offers a more comprehensive understanding of the interactions between bioactive molecules and cellular pathways [17]. Besides, the deep learning methodology has been an instrumental tool to direct studies in pharmacology, aspects involving the prediction of drug-target interaction (DTI), the repurposing of drugs, and the discovery of new drugs [18]. Indeed, DTI stands as one of the most direct and potent approaches for uncovering active compounds and their targets, which is critical for biomedical drug discovery and development [19, 20]. Traditional methods for predicting DTI have encountered financial and technological constraints, whereas computational approaches have demonstrated their effectiveness in achieving the same results [21]. Among these, deep learning serves as a potent *in silico* tool where numerous models are constructed to predict DTI [21]. DeepPurpose is a deep learning method offering a platform that integrates more than 50 sophisticated deep learning models, 15 drug encodings, and 7 target encodings based on multiple databases (DAVIS, KIBA, *etc*.) [22]. The models utilized in DeepPurpose have been indicated to attain leading-edge predictive capacity on DTI benchmark datasets [22]. Therefore, we employed network pharmacology analysis to investigate the anti-T2DM mechanisms of dapagliflozin and used the DeepPurpose algorithm to verify the binding activity of dapagliflozin with identified key targets. This study aimed to reveal the pharmacological mechanisms of dapagliflozin against T2DM and provide novel targets for the treatment of T2DM.

## 2 MATERIALS AND METHODS

### 2.1 Screening of Dapagliflozin-associated Targets

Targets related to dapagliflozin were collected from Comparative Toxicogenomics Database (CTD; http://ctdbase.org/) [23], SwissTargetPrediction (http://swisstargetprediction.ch/) [24], and SuperPred (version 3.0; https://prediction.charite.de/index.php) [25] databases. The species was set to *Homo sapiens*. The searching results were merged and the duplicate targets were deleted. Standardization of the target names was conducted using the UniProt database (http://www.uniprot.org/) [26].

### 2.2 Screening of T2DM-associated Targets

Targets related to T2DM were searched from GeneCards (version 3.0; https://www.genecards.org/) [27] and DigSee (version 2.01; http://210.107.182.61/geneSearch/) [28] databases. Similarly, the species was set to *Homo sapiens*. For the retrieval results from GeneCards, the targets belonging to “protein-coding” were primarily selected, followed by the targets with a relevance score exceeding the median value. Finally, the targets of T2DM were determined after duplication deletion and UniProt standardization.

### 2.3 Identification of Potential Targets of Dapagliflozin against T2DM

The overlapping targets between the targets of dapagliflozin and T2DM were identified by running VennDiagram (version 1.7.3, http://bioinfogp.cnb.csic.es/tools/venny/index.html) [29]. The identified overlapping targets were regarded as the potential targets of dapagliflozin against T2DM and used for subsequent analyses. Additionally, the network of the overlapping targets was built using Cytoscape (version 3.8.2).

### 2.4 Gene Ontology (GO) Functional and Kyoto Encyclopaedia of Genes and Genomes (KEGG) Pathway Enrichment Analyses

GO functional and KEGG pathway enrichment analyses of the overlapping targets were conducted using clusterProfiler (http://bioconductor.org/packages/release/bioc/html/clusterProfiler.html) [30]. GO enrichment analysis covered three aspects: biological process (BP), cellular component (CC), and molecular function (MF). A hypergeometric distribution model was employed to determine the relevance of a specific GO term or KEGG pathway to the query genes using the following formula:


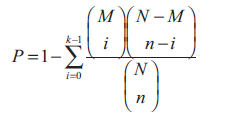


Where *N* is the total number of reference genes; *M* is the number of annotated genes in a GO and KEGG pathway; *n* is the number of the query genes; *k* is the number of genes shared by the reference and query gene sets. *P* value < 0.05 indicates a significant correlation.

### 2.5 Establishment of Protein-protein Interaction (PPI) Network and Identification of Pivotal Targets

A PPI network was built by entering the overlapping targets into the Retrieval of Interacting Genes/Proteins (STRING; https://string-db.org/) [31] database and visualized by Cytoscape. The species was set to *Homo sapiens* and the confidence score was set to > 0.4. Then, the degree, maximum neighborhood component (MNC), and edge percolated component (EPC) algorithms of CytoHubba plug-in in Cytoscape were employed to select the top 30 targets from the PPI network, respectively. Finally, the targets enriched in the PI3K-Akt signaling pathway (revealed by KEGG pathway enrichment analysis) were intersected with the top 30 targets screened by the above three algorithms using VennDiagram to obtain the pivotal anti-T2DM targets of dapagliflozin. Besides, the circle tree of the BPs in which the pivotal targets were enriched was mapped by Cytoscape.

### 2.6 Assessment of the Binding Affinity of Dapagliflozin with the Key Targets *via* DeepPurpose

DeepPurpose (https://github.com/kexinhuang12345/DeepPurpose), a deep learning library for DTI prediction, facilitates swift model building through a coding platform [22]. This platform integrates more than 50 deep learning models, seven protein encoding mechanisms, and eight compound encoding methods [22]. In the present study, DeepPurpose was employed to evaluate the interaction between dapagliflozin and the key targets. DeepPurpose took the pairs consisting of the simplified molecular-input line-entry system (SMILES) string for dapagliflozin and the amino acid sequences of the target proteins as input. Subsequently, these input data were introduced to molecular encoders, which define an intricate transformation function responsible for converting compounds and proteins into a vector representation [22]. For dapagliflozin, the encoder, Multi-Layer Perceptrons (MLP) on Morgan, was used; for amino acid sequences of the target proteins, the encoder, MLP on Amino Acid Composition (AAC) was used. Finally, the binding scores of dapagliflozin with the key targets were calculated using Morgan_AAC_DAVIS model. The SMILES of dapagliflozin was acquired from PubChem database (https://pubchem.ncbi.nlm.nih.gov/), and all amino acid sequences of the key targets were obtained from the UniProt database (https://www.uniprot.org/).

## 3 RESULTS

### 3.1 Determination of Potential anti-T2DM Targets of Dapagliflozin

**Fig. (1):**
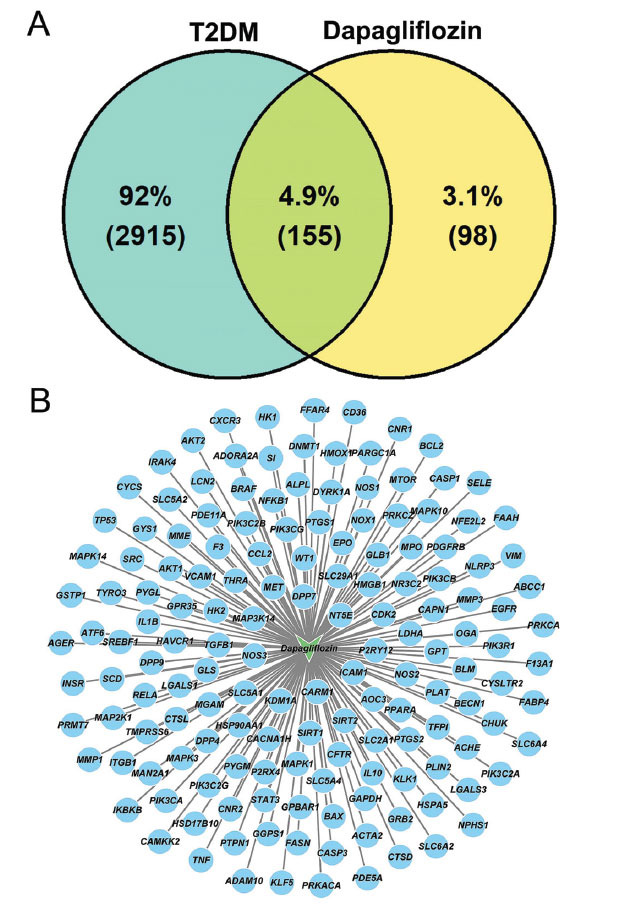
Determination of potential anti-T2DM targets of dapagliflozin. (**A**) Venn diagram of overlapping targets of dapagliflozin and T2DM. (**B**) Dapagliflozin- overlapping target network. T2DM, type 2 diabetes mellitus.

After searching CTD, SwissTargetPrediction, and SuperPred databases, we collected 263 targets related to dapagliflozin in total. Then, 253 targets of dapagliflozin were acquired following duplication removal and UniProt standardization. At the same time, 3,070 targets associated with T2DM were obtained from GeneCards and DigSee databases. Finally, we acquired 155 overlapping targets by taking the intersection of the 253 dapagliflozin-related and 3,070 T2DM-related targets in VennDiagram (Fig. **1A** and Table **1**). The 155 overlapping targets were deemed as the potential anti-T2DM targets of dapagliflozin and further analyzed. Besides, the network of dapagliflozin and these overlapping targets was exhibited in Fig. (**1B**).

### 3.2 GO Functional and KEGG Pathway Enrichment Analyses

**Fig. (2):**
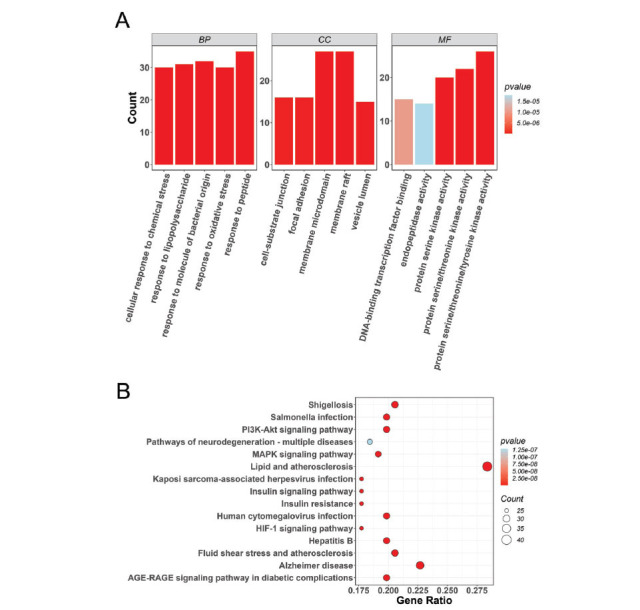
GO functional and KEGG pathway enrichment analyses. (**A**) Top 5 significantly enriched BPs, CCs, and MFs. (**B**) Top 15 significantly enriched KEGG pathways. *P* value < 0.05. GO, Gene Ontology; KEGG, Kyoto Encyclopedia of Genes and Genomes; BP, biological process; CC, cellular component; MF, molecular function.

GO functional and KEGG pathway enrichment analyses were performed to figure out the possible mechanisms by which dapagliflozin acts on the 155 targets to exert anti-T2DM effects. In this study, we obtained 2,215 significantly enriched GO terms (including 2,019 BPs, 48 CCs, and 148 MFs; Table **S1**) and 167 KEGG pathways (Table **S2**), respectively (*p* value < 0.05). Furthermore, the top 5 BPs, CCs, and MFs, as well as the top 15 KEGG pathways, were selected according to the count value. Response to the peptide (GO: 1901652; count = 35), membrane microdomain (GO: 0098857; count = 27), and protein serine/threonine/tyrosine kinase activity (GO: 0004712; count = 26) were included in the top 5 BPs, CCs, and MFs, respectively (Fig. **2A** and Table **2**). Meanwhile, multiple signaling pathways, such as the PI3K-Akt signaling pathway (hsa04151; count = 28), and MAPK signaling pathway (hsa04010; count = 27), and the AGE-RAGE signaling pathway in diabetic complications (hsa04933; count = 28) were among the top 15 KEGG pathways (Fig. **2B** and Table **3**). As a result, the PI3K-Akt signaling pathway was selected as the key pathway in the anti-T2DM mechanisms of dapagliflozin and used for further analyses.

### 3.3 PPI Network Analysis and Determination of Key Targets

**Fig. (3):**
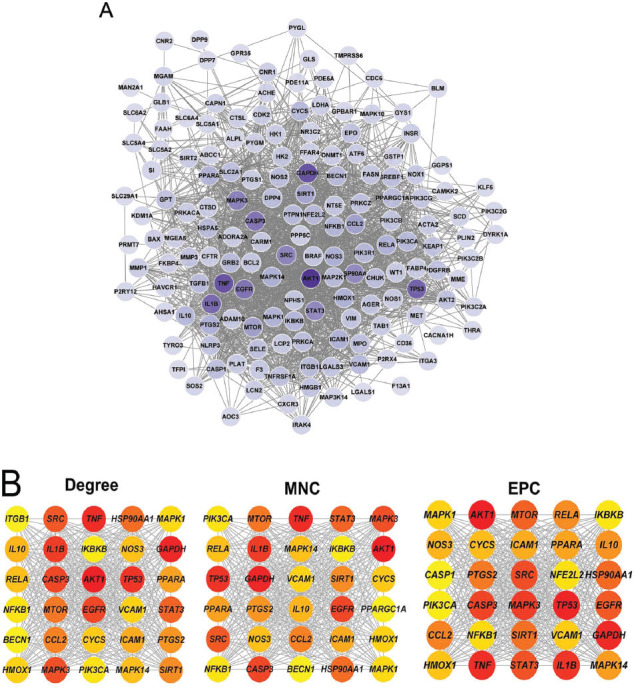
PPI network analysis and screening of hub targets. (**A**) PPI network. (**B**) Top 30 targets screened by the degree, MNC, and EPC algorithms of cytoHubba. PPI, protein-protein interaction; MNC, maximum neighborhood component; EPC, edge percolated component.

**Fig. (4):**
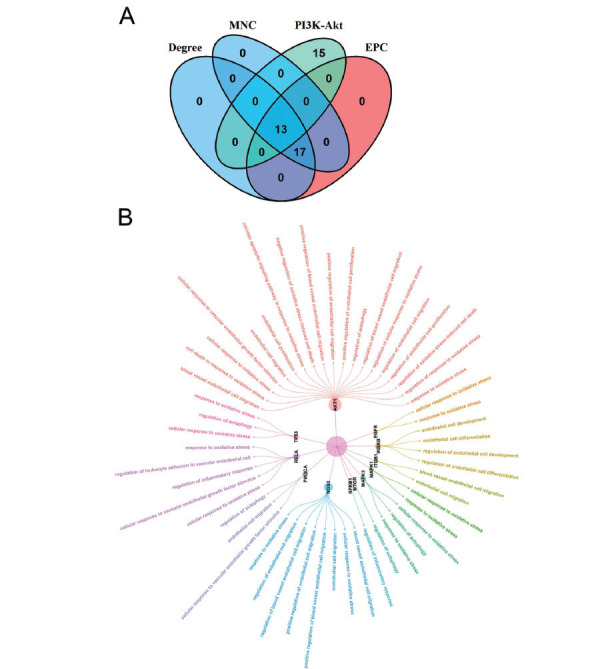
Determination of key anti-T2DM targets of dapagliflozin. (**A**) Venn diagram of the targets enriched in PI3K-Akt signaling pathway and the top 30 targets screened by the degree, MNC, and EPC algorithms. (**B**) Circle tree of the significant BPs in which the key targets were enriched. *P* value < 0.05. T2DM, type 2 diabetes mellitus; MNC, maximum neighborhood component; EPC, edge percolated component; BP, biological process.

A PPI network was constructed to clarify the correlations among the 155 overlapping targets. Here, the PPI network was composed of 162 nodes and 2,084 edges (score > 0.4; Fig. **3A**). Subsequently, we acquired the top 30 targets *via* the degree, MNC, and EPC algorithms, respectively (Fig. **3B**). Finally, we obtained 13 overlapping targets by intersecting the 28 targets enriched in the PI3K-Akt signaling pathway with the top 30 targets screened by the above three algorithms (Fig. **4A**). The 13 overlapping target genes were AKT serine/threonine kinase 1 (AKT1), phosphatidylinositol-4,5-bisphosphate 3-kinase catalytic subunit alpha (PIK3CA), nitric oxide synthase 3 (NOS3), epidermal growth factor receptor (EGFR), mitogen-activated protein kinase 1 (MAPK1), mitogen-activated protein kinase 3 (MAPK3), heat shock protein 90 alpha family class A member 1 (HSP90AA1), mechanistic target of rapamycin kinase (MTOR), RELA proto-oncogene, NF-kB subunit (RELA), nuclear factor kappa B subunit 1 (NFKB1), an inhibitor of nuclear factor kappa B kinase subunit beta (IKBKB), and integrin subunit beta 1 (ITGB1), and tumor protein p53 (TP53). Notably, we found that the 13 key targets were primarily enriched in OS-, endothelial cell-, and autophagy-related BPs, such as cellular response to oxidative stress, endothelial cell migration, and regulation of autophagy (Fig. **4B** and Table **4**).

### 3.4 Determination of Dapagliflozin-key Target Interactions through DeepPurpose

**Fig. (5):**
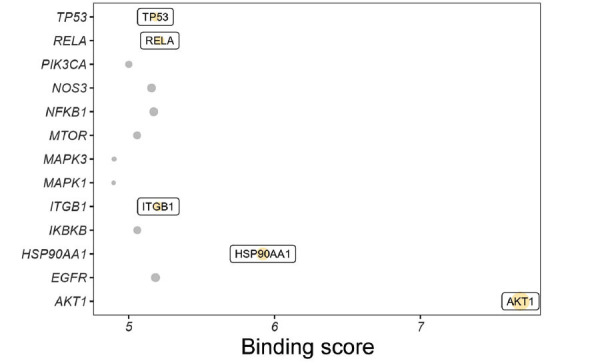
Determination of dapagliflozin-key target interactions by DeepPurpose.

DeepPurpose was employed to measure the affinity of dapagliflozin with the key targets (AKT1, PIK3CA, NOS3, EGFR, MAPK1, MAPK3, HSP90AA1, MTOR, RELA, NFKB1, IKBKB, ITGB1, and TP53). Here, we found that dapagliflozin had the optimal binding affinity with AKT1, HSP90AA1, RELA, ITGB1, and TP53, and the binding scores were 7.686, 5.92, 5.2133, 5.2036, and 5.1842, respectively (Fig. **5** and Table **5**). Among these, the binding score of dapagliflozin with AKT1 was the highest, indicating a strong interaction between them.

## 4 DISCUSSION

T2DM presents a significant public health challenge, which causes substantial health and societal burden and is related to early mortality and morbidity [32]. Dapagliflozin, a highly effective, reversible, and selective SGLT2 inhibitor, is globally recognized for the treatment of T2DM [10]. In the present study, a network pharmacology approach combined with deep learning technology was used to explore the underlying mechanisms of dapagliflozin against T2DM and identify the key therapeutic targets.

By retrieving public databases, we obtained 253 dapagliflozin-related and 3,070 T2DM-related targets. As a consequence, 155 overlapping targets of dapagliflozin and T2DM were determined, which were regarded as the potential anti-T2DM of dapagliflozin.

Subsequently, to further ascertain the mechanisms of dapagliflozin treating T2DM by acting on the 155 potential targets, we carried out GO functional and KEGG pathway enrichment analyses. GO enrichment analysis revealed that the 155 targets were primarily associated with response to peptide, membrane microdomain, and protein serine/threonine/tyrosine kinase activity, *etc*. KEGG pathway enrichment analysis showed that several signaling pathways, such as the PI3K-Akt signaling pathway, MAPK signaling pathway, and AGE-RAGE signaling pathway in diabetic complications, were implicated in the anti-T2DM mechanisms of dapagliflozin. The PI3K-Akt pathway serves as the crucial hub for insulin signaling transduction, and it regulates a range of cellular activities, such as blood glucose absorption, cellular metabolism, cell survival, proliferation, and glycogen production [33]. Accumulating evidence shows that PI3K-Akt signaling plays a crucial role in the development and progression of T2DM [34, 35]. Interestingly, a recent study has indicated that dapagliflozin can improve β cell function in a mouse model of T2DM, which might be partly due to its regulatory role in the PI3K-Akt axis [36]. The MAPK family triggers a series of cascades that modulate multiple biological activities in reaction to diverse cellular signals, including insulin signaling [37]. The MAPK pathway consists of JNK (c-Jun N-terminal kinase), ERK1/2 (extracellular signal-regulated kinases 1/2), and p38 (p38 kinase) [37]. A previous study demonstrated that MAPK/ ERK signaling might participate in the regulation of β cell function [38]. Studies reveal that AGE-RAGE signaling is implicated in the development of T2DM and its complications [39, 40]. The formation and accumulation of AGEs is an especially significant outcome of T2DM [41]. AGEs can lead to inflammation by interacting with their receptors (RAGEs) [39]. Also, the interaction of AGEs with RAGEs is linked to peripheral insulin resistance, resulting in the initiation of signaling cascades that activate the JNK pathway, IKKα/β, and the key transcription factor, NF-κB [42]. In short, these findings indicate the involvement of multiple pathways and mechanisms in the anti-T2DM process of dapagliflozin, and the PI3K-Akt signaling pathway was chosen for further analysis.

Through the degree, MNC, and EPC algorithms of CytoHubba, the top 30 targets were respectively selected from the PPI network. Consequently, we identified AKT1, PIK3CA, NOS3, EGFR, MAPK1, MAPK3, HSP90AA1, MTOR, RELA, NFKB1, IKBKB, ITGB1, and TP53 as the key anti-T2DM targets of dapagliflozin by intersecting the 28 targets enriched in PI3K-Akt signaling pathway with the top 30 targets screened by the three algorithms. Furthermore, these key targets were mainly associated with oxidative stress-, endothelial cell-, and autophagy-related BPs.

Finally, the deep learning method, DeepPurpose, was used to evaluate the binding affinity of dapagliflozin with the key targets. DeepPurpose is an accessible and comprehensive deep learning library for DTI prediction, which is vital for drug discovery [22]. Results showed that dapagliflozin had comparatively good binding affinity with AKT1, HSP90AA1, RELA, ITGB1, and TP53, among which, dapagliflozin showed the strongest binding activity with dapagliflozin. AKT, a member of the serine/threonine kinase family, participates in signaling pathways related to cell proliferation, apoptosis, angiogenesis, migration, glucose metabolism, and others [43]. Belonging to the AKT family, AKT1 functions as an anti-apoptotic signaling kinase in various cells [44]. A previous study indicated that AKT1 polymorphisms might be associated with the risk of Alzheimer's disease in patients with T2DM [45]. In line with our findings, a recent bioinformatics analysis has indicated a good binding affinity between dapagliflozin and AKT1 [46]. Being a stress-inducible protein, HSP90AA1 acts as a chaperone of misfolded proteins, responsible for maintaining cellular homeostasis. HSP90AA1 modulates the stability and functionality of protein kinases, transcription factors, and various proteins involved in cell signaling pathways [47]. Accumulating evidence revealed that HSP90AA1 has significant implications for autophagy [48, 49]. A recent study has indicated that activating the HSP90AA1-Akt pathway can alleviate inflammation and apoptosis in an *in vitro* model of acute kidney injury [50]. RELA (also known as p65) is a crucial member of the NF-κB family that serves as a key mediator of inflammation [51]. A previous study found increased phosphorylated p65 in T2DM rats, which might be related to dysregulated MAPK/PI3K/Akt signaling [52]. Interestingly, a recent investigation has shown that dapagliflozin can reduce the shift of p65 to the nucleus, which can be beneficial for the suppression of STZ-induced cardiac hypertrophy in a rat model of T2DM [53]. This further suggests that dapagliflozin might act on RELA in the anti-T2DM process. As the largest subfamily of integrins, ITGB1 is widely recognized as a cell membrane receptor that plays a crucial role in various physiological and pathological processes. Integrins are a class of receptors for heterodimeric extracellular matrix that function in cell-matrix adhesion, intracellular signaling, and regulation of cell proliferation, differentiation, and survival [54]. A previous investigation revealed the regulatory role of active integrins in insulin sensitivity in adipocytes and systemic metabolism [55]. Another study observed a significant decrease in the protein expression of alpha II B integrin in the serum samples of patients with T2DM following dapagliflozin treatment [56]. Furthermore, it was indicated that unstable ITGB1 mRNA could inhibit inflammation and neovascularization to prevent the progression of diabetic retinopathy (a main complication of DM) in a mouse model [57]. TP53, a transcription factor, reacts to diverse cellular stresses and modulates target genes implicated in various cellular processes, such as apoptosis, DNA repair, and metabolism [58]. Increasingly, studies suggest that TP53 plays a crucial role in the onset and development of T2DM [58-60]. Recent evidence has shown that TP53 can serve as a key target of dapagliflozin against anemia in elderly patients with heart failure [46]. Given that the coexistence of heart failure and T2DM is prevalent [61], it is reasonable to assume that dapagliflozin might affect TP53 in the treatment of T2DM. Collectively, AKT1, HSP90AA1, RELA, ITGB1, and TP53 might serve as the foremost targets of dapagliflozin against T2DM.

This study has certain limitations. Firstly, this study largely relied on *in silico* analyses and bioinformatics approaches without *in vitro* or *in vivo* experimental validation. Secondly, the crosstalk among oxidative stress, endothelial dysfunction, and autophagy in the anti-T2DM mechanisms remains to be expounded. Thirdly, we focused on the identification of PI3K-Akt pathway-related targets, which may neglect other significant targets for the treatment of T2DM. In our subsequent studies, both *in vitro* and *in vivo* experiments will be conducted to explore the specific regulatory role of dapagliflozin in the PI3K-Akt pathway and key targets against T2DM.

## CONCLUSION

This study revealed that AKT1, HSP90AA1, RELA, ITGB1, and TP53 were the foremost targets of dapagliflozin treating T2DM through the PI3K-Akt pathway. Besides, dapagliflozin might alleviate T2DM by regulating oxidative stress, endothelial function, and autophagy. This study provides novel anti-T2DM targets, sheds light on the associated mechanisms and pathways, and furnishes a foundation for further research into the molecular mechanisms and the treatment of T2DM.

## Figures and Tables

**Table 1:** Overlapping targets of dapagliflozin and T2DM.

**Gene Name**	**Gene Name**	**Gene Name**	**Gene Name**
ABCC1	EPO	MAP2K1	PRKACA
ACHE	F13A1	MAP3K14	PRKCA
ACTA2	F3	MAPK1	PRKCZ
ADAM10	FAAH	MAPK10	PRMT7
ADORA2A	FABP4	MAPK14	PTGS1
AGER	FASN	MAPK3	PTGS2
AKT1	FFAR4	MET	PTPN1
AKT2	GAPDH	MGAM	PYGL
ALPL	GGPS1	MME	PYGM
AOC3	GLB1	MMP1	RELA
ATF6	GLS	MMP3	SCD
BAX	GPBAR1	MPO	SELE
BCL2	GPR35	MTOR	SI
BECN1	GPT	NFE2L2	SIRT1
BLM	GRB2	NFKB1	SIRT2
BRAF	GSTP1	NLRP3	SLC29A1
CACNA1H	GYS1	NOS1	SLC2A1
CAMKK2	HAVCR1	NOS2	SLC5A1
CAPN1	HK1	NOS3	SLC5A2
CARM1	HK2	NOX1	SLC5A4
CASP1	HMGB1	NPHS1	SLC6A2
CASP3	HMOX1	NR3C2	SLC6A4
CCL2	HSD17B10	NT5E	SRC
CD36	HSP90AA1	OGA	SREBF1
CDK2	HSPA5	P2RX4	STAT3
CFTR	ICAM1	P2RY12	TFPI
CHUK	IKBKB	PDE11A	TGFB1
CNR1	IL10	PDE5A	THRA
CNR2	IL1B	PDGFRB	TMPRSS6
CTSD	INSR	PIK3C2A	TNF
CTSL	IRAK4	PIK3C2B	TP53
CXCR3	ITGB1	PIK3C2G	TYRO3
CYCS	KDM1A	PIK3CA	VCAM1
CYSLTR2	KLF5	PIK3CB	VIM
DNMT1	KLK1	PIK3CG	WT1
DPP4	LCN2	PIK3R1	-
DPP7	LDHA	PLAT	-
DPP9	LGALS1	PLIN2	-
DYRK1A	LGALS3	PPARA	-
EGFR	MAN2A1	PPARGC1A	-

**Table 2:** Top 5 significantly enriched BP, CC, and MF terms.

**Ontology**	**ID**	**Description**	***p* Value**	**Count**
BP	GO:1901652	Response to peptide	4.56E-23	35
BP	GO:0002237	Response to molecule of bacterial origin	2.86E-24	32
BP	GO:0032496	Response to lipopolysaccharide	6.63E-24	31
BP	GO:0062197	Cellular response to chemical stress	9.14E-23	30
BP	GO:0006979	Response to oxidative stress	1.88E-19	30
CC	GO:0045121	Membrane raft	5.33E-20	27
CC	GO:0098857	Membrane microdomain	5.77E-20	27
CC	GO:0005925	Focal adhesion	2.27E-07	16
CC	GO:0030055	Cell-substrate junction	3.02E-07	16
CC	GO:0031983	Vesicle lumen	5.22E-08	15
MF	GO:0004712	Protein serine/threonine/tyrosine kinase activity	6.40E-15	26
MF	GO:0004674	Protein serine/threonine kinase activity	1.23E-11	22
MF	GO:0106310	Protein serine kinase activity	2.62E-11	20
MF	GO:0140297	DNA-binding transcription factor binding	1.06E-05	15
MF	GO:0004175	Endopeptidase activity	1.78E-05	14

**Table 3:** Top 15 significantly enriched KEGG pathways.

**ID**	**Description**	***p* Value**	**Count**
hsa05417	Lipid and atherosclerosis	4.64E-31	40
hsa05010	Alzheimer disease	4.46E-14	32
hsa05418	Fluid shear stress and atherosclerosis	5.93E-24	29
hsa05131	Shigellosis	9.90E-17	29
hsa04933	AGE-RAGE signaling pathway in diabetic complications	5.17E-27	28
hsa05161	Hepatitis B	8.97E-21	28
hsa05163	Human cytomegalovirus infection	7.69E-17	28
hsa05132	*Salmonella* infection	1.12E-15	28
hsa04151	PI3K-Akt signaling pathway	8.01E-12	28
hsa04010	MAPK signaling pathway	5.81E-13	27
hsa05022	Pathways of neurodegeneration - multiple diseases	1.28E-07	26
hsa04931	Insulin resistance	6.62E-22	25
hsa04066	HIF-1 signaling pathway	8.48E-22	25
hsa04910	Insulin signaling pathway	3.34E-19	25
hsa05167	Kaposi sarcoma-associated herpesvirus infection	1.84E-15	25

**Table 4:** Significant BP terms the key targets mainly enriched in.

**Gene Name**	**BP Term**
AKT1	Blood vessel endothelial cell migration
ITGB1	Blood vessel endothelial cell migration
NOS3	Blood vessel endothelial cell migration
AKT1	Cell death in response to oxidative stress
AKT1	Cellular response to oxidative stress
EGFR	Cellular response to oxidative stress
MAPK1	Cellular response to oxidative stress
MAPK3	Cellular response to oxidative stress
NOS3	Cellular response to oxidative stress
RELA	Cellular response to oxidative stress
TP53	Cellular response to oxidative stress
AKT1	Cellular response to vascular endothelial growth factor stimulus
PIK3CA	Cellular response to vascular endothelial growth factor stimulus
RELA	Cellular response to vascular endothelial growth factor stimulus
IKBKB	Endothelial cell development
IKBKB	Endothelial cell differentiation
AKT1	Endothelial cell migration
ITGB1	Endothelial cell migration
NOS3	Endothelial cell migration
PIK3CA	Endothelial cell migration
AKT1	Endothelial cell proliferation
AKT1	Intrinsic apoptotic signaling pathway in response to oxidative stress
AKT1	Negative regulation of oxidative stress-induced cell death
AKT1	Positive regulation of blood vessel endothelial cell migration
NOS3	Positive regulation of blood vessel endothelial cell migration
AKT1	Positive regulation of endothelial cell migration
NOS3	Positive regulation of endothelial cell migration
AKT1	Positive regulation of endothelial cell proliferation
AKT1	Regulation of autophagy
MAPK3	Regulation of autophagy
MTOR	Regulation of autophagy
PIK3CA	Regulation of autophagy
TP53	Regulation of autophagy
AKT1	Regulation of blood vessel endothelial cell migration
NOS3	Regulation of blood vessel endothelial cell migration
AKT1	Regulation of cellular response to oxidative stress
IKBKB	Regulation of endothelial cell development
IKBKB	Regulation of endothelial cell differentiation
AKT1	Regulation of endothelial cell migration
NOS3	Regulation of endothelial cell migration
AKT1	Regulation of endothelial cell proliferation
NFKB1	Regulation of inflammatory response
RELA	Regulation of inflammatory response
RELA	Regulation of leukocyte adhesion to vascular endothelial cell
AKT1	Regulation of oxidative stress-induced cell death
AKT1	Regulation of response to oxidative stress
AKT1	Response to oxidative stress
EGFR	Response to oxidative stress
MAPK1	Response to oxidative stress
MAPK3	Response to oxidative stress
NOS3	Response to oxidative stress
RELA	Response to oxidative stress
TP53	Response to oxidative stress

**Table 5:** Dapagliflozin-key target interactions.

**Compound**	**PubChem ID**	**Target**	**UniProt ID**	**Score**
Dapagliflozin	9887712	AKT1	P31749	7.686
		HSP90AA1	P07900	5.92
		RELA	Q04206	5.2133
		ITGB1	P05556	5.2036
		TP53	P04637	5.1842
		EGFR	P00533	5.1841
		NFKB1	P19838	5.1717
		NOS3	P29474	5.1574
		IKBKB	O14920	5.0596
		MTOR	P42345	5.0583
		PIK3CA	P42336	5.0008
		MAPK3	P27361	4.9007
		MAPK1	P28482	4.8974

## Data Availability

The data that support the findings of this study are available from the corresponding author, [YD], on special request.

## LIST OF ABBREVIATIONS

AAC: Amino Acid Composition
AHAs: Antihyperglycaemic Agents
AKT1AKT: Serine/Threonine Kinase 1
BP: Biological Process
CC: Cellular Component
CTD: Comparative Toxicogenomics Database
DM: Diabetes Mellitus
DTI: Drug-target Interaction
EPC: Edge Percolated Component
GO: Gene Ontology
KEGG: Kyoto Encyclopaedia of Genes and Genomes
MCC: Maximal Clique Centrality
MF: Molecular Function
MLP: Multi-Layer Perceptrons
MNC: Maximum Neighborhood Component
NOS3: Nitric Oxide Synthase 3
PIK3CA: Phosphatidylinositol-4,5-bisphosphate 3-kinase Catalytic Subunit Alpha
PPI: Protein-protein Interaction
SGLT2: Sodium-glucose Cotransporter-2
SMILES: Simplified Molecular-input Line-entry System
T2DM: Type 2 Diabetes Mellitus
