# Supplementary material for: Exploration of Pharmacological Mechanisms of Dapagliflozin against Type 2 Diabetes Mellitus through PI3K-Akt Signaling Pathway based on Network Pharmacology Analysis and Deep Learning Technology
Source: Curr Comput Aided Drug Des. 2024 Jan 9;21(4):452–65. doi: 10.2174/0115734099274407231207070451 (PMC12272070; doi:10.2174/0115734099274407231207070451)
Supplement: Supplementary file 1 [file CCADD-21-4-452_SD1.zip › CCADD-21-4-452_SD1/CCADD-21-4-03-Supply.pdf]

SUPPLEMENTARY MATERIAL

Exploration of Pharmacological Mechanisms of Dapagliflozin against Type 2 Diabetes Mellitus through PI3K-Akt Signaling Pathway based on Network Pharmacology Analysis and Deep Learning Technology

Jie Wu[1,#], Yufan Chen[2,#], Shuai Shi[3], Junru Liu[4], Fen Zhang[1], Xingxing Li[1], Xizhi Liu[1], Guoliang Hu[5] and Yang Dong[1,*]

[1]Department of Cardiology, Jinhua People's Hospital, Jinhua, Zhejiang, China; [2]Department of Blood Donation Service, Central Blood Station of Jinhua, Jinhua, Zhejiang, China; [3]Department of IVF, Jinhua People's Hospital, Jinhua, Zhejiang, China; [4]Department of Endocrinology, Jinhua People's Hospital, Jinhua, Zhejiang, China; [5]Department of Ultrasound in Medicine, Jinhua People's Hospital, Jinhua, Zhejiang, China
